# Supplementary material for: Neuroinflammation, body temperature and behavioural changes in CD1 male mice undergoing acute restraint stress: An exploratory study
Source: PLoS One. 2021 Nov 15;16(11):e0259938. doi: 10.1371/journal.pone.0259938 (PMC8592432; doi:10.1371/journal.pone.0259938)
Supplement: Attachment — Submitted filename: Response to Reviewers.docx [file pone.0259938.s001.docx]

Please pay particular attention to the request of reviewer 1 to provide additional controls for data analysis and interpretation.

Reviewer's Responses to Questions

**Comments to the Author**

1. If the authors have adequately addressed your comments raised in a previous round of review and you feel that this manuscript is now acceptable for publication, you may indicate that here to bypass the “Comments to the Author” section, enter your conflict of interest statement in the “Confidential to Editor” section, and submit your "Accept" recommendation.

Reviewer #1: (No Response)

Reviewer #2: (No Response)

2. Is the manuscript technically sound, and do the data support the conclusions?

The manuscript must describe a technically sound piece of scientific research with data that supports the conclusions. Experiments must have been conducted rigorously, with appropriate controls, replication, and sample sizes. The conclusions must be drawn appropriately based on the data presented.

Reviewer #1: Partly

Reviewer #2: Yes

3. Has the statistical analysis been performed appropriately and rigorously?

Reviewer #1: Yes

Reviewer #2: Yes

4. Have the authors made all data underlying the findings in their manuscript fully available?

The [PLOS Data policy](https://eur03.safelinks.protection.outlook.com/?url=http%3A%2F%2Fwww.plosone.org%2Fstatic%2Fpolicies.action%23sharing&data=04%7C01%7Cjohnny.roughan%40newcastle.ac.uk%7C4c7000a4277e408c99eb08d93a28d9a1%7C9c5012c9b61644c2a91766814fbe3e87%7C1%7C0%7C637604770329967972%7CUnknown%7CTWFpbGZsb3d8eyJWIjoiMC4wLjAwMDAiLCJQIjoiV2luMzIiLCJBTiI6Ik1haWwiLCJXVCI6Mn0%3D%7C1000&sdata=ZVjHgZ4Wj1uf89pMTnuvezBwXrbSkVM%2B2ib7p8OlZeM%3D&reserved=0) requires authors to make all data underlying the findings described in their manuscript fully available without restriction, with rare exception (please refer to the Data Availability Statement in the manuscript PDF file). The data should be provided as part of the manuscript or its supporting information, or deposited to a public repository. For example, in addition to summary statistics, the data points behind means, medians and variance measures should be available. If there are restrictions on publicly sharing data—e.g. participant privacy or use of data from a third party—those must be specified.

Reviewer #1: No

Reviewer #2: Yes

5. Is the manuscript presented in an intelligible fashion and written in standard English?

PLOS ONE does not copyedit accepted manuscripts, so the language in submitted articles must be clear, correct, and unambiguous. Any typographical or grammatical errors should be corrected at revision, so please note any specific errors here.

Reviewer #1: Yes

Reviewer #2: No

6. Review Comments to the Author

Please use the space provided to explain your answers to the questions above. You may also include additional comments for the author, including concerns about dual publication, research ethics, or publication ethics. (Please upload your review as an attachment if it exceeds 20,000 characters)

Reviewer #1: I thank the authors for the improved manuscript. Figure 1 is helpful in interpreting the experiment conducted.

There are still a few points of concern:
1. NEW. It is unclear when the behavioral tests and samples were collected during the light phase of the day (0600 - 1800 h). Is it consistent? It makes a difference if it is close to the transitions (ie 0600 - ~ 0800 h and 1500 - ~1800 h) for the mice. Since this is a NC3R study, the authors should also state what time of the day the vivarium caretakers enter the room as this can also have an impact on the study.

*We thank the reviewer for highlighting this important detail.* *The time when the behavioral tests and samples were collected was consistently during the light phase,* *at least one hour away from the transition phase. The specific time of the day is reported in the “Experimental Design” paragraph, where the procedures performed in Day 2, 4 and 5 were described. In the vivarium, light was on at 6 am and off at 6pm. When necessary, caretakers entered the room always at the same hour which was around 9 am. This information is now in the manuscript in the* *“Experimental Design” paragraph (from line 214 of the Revised Manuscript with Track Changes).*

2. The authors made a very good counter argument about the very low baseline time in open arms (significantly less than 20-30%). However, the data does not reflect their argument. There should be parallel changes in open arm time and open arm entry. I am okay to waive the 20-30% baseline but the authors should show that the mice were actively exploring. In addition to the measures in Table 3A, it would be good to include activity in the open arm and activity in the closed arm. Other measures of exploration are head dip, sniffing etc.

*Thank you for acknowledging the validity of the counter argument in our initial reply. We agree that* *there should be an approximate correlation between the number of open arm entries and time spent on the open arms of the EPM. We cannot explain such low numbers of open arm entries but note that it is not unusual. For example, Henderson et al. reported an average of less than 1 open arm entry following tail-handling, increasing to only 3.5 in mice handled non-aversively (using a tunnel: https://doi.org/10.1038/s41598-020-71476-y). In their study the corresponding percentage of time mice in each handling group spent on the open arms was only 7 and 18%, respectively. In our study, ignoring the relatively minor effect that handling method had, the equivalent values were 5.6% before stress vs. 7.6 afterwards.* *We also checked to see if our data met the reviewers correct assertion that there should be parallel changes in open arm entries and open arm time. The proportion of entries vs. time was between 3-4 before handling and between 5 and 7 afterwards. We admit that these values are still low, but actually do parallel one another. Furthermore, and as we previously said, the behavioural outcomes were subsidiary to our main objective of exploring a possible neuroinflammatory effect of acute restraint under bright light. Handling method, and whether it might ameliorate the response to this stressor was secondary. Since there were not significant effects of this on the other parameter we evaluated (protected-stretched attend), we do not think it would be a sensible use of time to re-evaluate the activity of the mice on the open and closed arms, as we would likely arrive at the same conclusion – that handling had minimal effect. That’s said we do agree with the reviewer that these values are low, and have acknowledged this in the “Study Limitation” paragraph (from line 609 of the Revised Manuscript with Track Changes).*

3. Immunohistochemistry.
The 40X is an improvement. Thank you.
Regarding controls for the Iba1+ cells, the authors still did not address what controls they used. Controls for immunohistochemistry typically includes: 1. removal of primary antibody, 2. preabsorption of the antibody using a peptide (this should be easy to purchase or synthesize) or 3. use an Iba1 knockout mouse. The third option is idea but not always possible. Option #2 is the more commonly used option. The reason it is necessary to conduct a negative control is that immunohistochemistry is sensitive to many subtle changes in protocol - fixative, antigen retrieval etc etc etc. These subtle changes can cause changes to the epitope which can lead to false positives or partial positives/negatives.

*Thank you for acknowledging the improvement of the 40X figure. In order to have controls for immunohistochemistry we repeated reactions on a double set of slides of all brain samples, simultaneously, with Iba1 antibody and without it. The reactions on slides performed by removal of primary antibody did not show any signal. We have now specified the negative control used in the text (line 368) and modified figure 5 accordingly.*

4. NEW. More details are needed for where Iba1+ cells were counted. More details are needed to define the PVN and the entire section posterior to the midpoint of the brain. More typical details include: 1. using a stereotaxic atlas to provide the coordinates (rostro-caudal and lateral) for what you are looking at and 2. a photomicrograph of the region of interest that is outlined. In addition, more information is also needed to how the authors measured the Iba+ cells in a non-biased manner.

*Thanks for the comment. We used the following atlas: Paxinos G. and Franklin, K.B.J., The mouse brain in stereotaxic coordinates, Academic Press, New York, 2001, ISBN 0-12-547637-X, in order to define the brain areas. The entire brain section posterior to the midpoint was taken 2 mm posterior to the bregma. According to Paxinos Atlas, we identify PVN just beneath the 3^rd^ ventricle, at the following coordinates: -1.5 rostro-caudal; +/- 0.1 lateral (i.e. millimeters from bregma and from the midline, respectively). Atlas information and coordinates are now provided in the text (from line 372) and the photomicrograph of the region of interest that is outlined (PVN) is now in the modified fig. 5. Regarding information to how we measured the Iba+ cells in a non-biased manner, we provided a detailed description of our use of ImageJ software in the method paragraph of our original submission (from line 372); in particular, we specified that signals were automatically counted. Nevertheless, Reviewer’s comment is important and we have now added a sentence accordingly, also specifying that researchers performing histology were blinded to the treatment (from line 379).*

5. NEW. In the response to reviewers, the authors state that "an image at high magnification (40X) will allow to make evident the well delineated signal in the cell body and in the dendrites." Microglia do not have dendrites.

*The Reviewer is right, thanks for highlighting this error in our previous response.*

Reviewer #2: Please ensure that the term sex is used throughout the manuscript not sex. Your mice have a sex, they do not have a gender.

*Thanks for highlighting this error, which we have corrected accordingly.*

Tables & Figures – with regard to presentation of the data. It would be best to present the data for the sentinels (true controls) in the first line.

*Thank you for the suggestion. We have now modified all tables and figures accordingly.*

If the data are presented as a figure it is not necessary to present a table also, for example Table 3b contains the same information as presented in figure 8.

*We thank for this comment; nevertheless, a complete presentation with both table and figure for the EPM parameters was necessary to address the Reviewer 1’s comments.*

Table 2 – data from TH animals were not included here, so perhaps you may wish to omit this line from the table to avoid confusion.

*The meaning of these values was that there were no differences. Omitting these values would be in our opinion more and not less confusing. To address the reviewer’s concern, however, we have tried to clarify this issue in the legend. Thank you for the suggestion.*

Figures 3 and 4 should be combined into one Figure.

*Thank you for this suggestion. We have now combined Figure 3 and figure 4, accordingly.*

The data for the nesting behavior has not been added in table or figure format.

*Thank you for the suggestion. A new table 5 with the data for the nesting behaviour is now present in the manuscript*

For the EPM it is not conventional to use the term fold increase. Rather choose a better descriptor, such as ratio of post/pre, or normalized to prestress baseline.

*We thank the Reviewer for this comment. The table 3b and the EPM figure actually showed a ratio of post/pre, and we have double checked this by repeating all calculations. We have now edited figures, tables and manuscript text accordingly.*

There remains a number of grammatical and typographical errors. The manuscript would benefit from editing by a native English speaker.

*Thank you for the comment,* *the native English speaker co-author Dr. Roughan has edited the manuscript.*

7. PLOS authors have the option to publish the peer review history of their article ([what does this mean?](https://eur03.safelinks.protection.outlook.com/?url=https%3A%2F%2Fjournals.plos.org%2Fplosone%2Fs%2Feditorial-and-peer-review-process%23loc-peer-review-history&data=04%7C01%7Cjohnny.roughan%40newcastle.ac.uk%7C4c7000a4277e408c99eb08d93a28d9a1%7C9c5012c9b61644c2a91766814fbe3e87%7C1%7C0%7C637604770329977933%7CUnknown%7CTWFpbGZsb3d8eyJWIjoiMC4wLjAwMDAiLCJQIjoiV2luMzIiLCJBTiI6Ik1haWwiLCJXVCI6Mn0%3D%7C1000&sdata=l6fCB0WoHFDMl4mIIDgv9B%2FM8ykJbiPNbNkHNRpdxQk%3D&reserved=0)). If published, this will include your full peer review and any attached files.

*Authors are agreed to publish the peer review history of their article*

If you choose “no”, your identity will remain anonymous but your review may still be made public.


**Do you want your identity to be public for this peer review?** For information about this choice, including consent withdrawal, please see our [Privacy Policy](https://eur03.safelinks.protection.outlook.com/?url=https%3A%2F%2Fwww.plos.org%2Fprivacy-policy&data=04%7C01%7Cjohnny.roughan%40newcastle.ac.uk%7C4c7000a4277e408c99eb08d93a28d9a1%7C9c5012c9b61644c2a91766814fbe3e87%7C1%7C0%7C637604770329977933%7CUnknown%7CTWFpbGZsb3d8eyJWIjoiMC4wLjAwMDAiLCJQIjoiV2luMzIiLCJBTiI6Ik1haWwiLCJXVCI6Mn0%3D%7C1000&sdata=S4jkGRj0ZQajp4%2BIr0%2FGjGF2IwQeeXyuy1YQertmUDc%3D&reserved=0).

Reviewer #1: No

Reviewer #2: No
